# Co-existence of Different Forms of Skin Diseases

**Published:** 1883-08

**Authors:** 


ON THE CO-EXISTENCE OR SEQUENCE OF DIFFERENT FORMS OF SKIN DISEASES.—Dr. J. K. Spender, of the Bath Mineral Water Hospital, describes a "Case of Lichen Psoriasis," at that hospital, which occurred in a man aged 44. The symptoms of psoriasis soon disappeared under hydro-therapeutic treatment, but the battery had little control over that part of the eruption which might be correctly termed lichen. The author continues: "I have had lately, in private practice, a case which illustrates the way in which accidental circumstances may cause different forms of skin-diseases to succeed each other. A retired farmer, aged 80, very temperate in all his ways, became depressed and generally out of health at the beginning of this January. In a few days, there was an acute eruption of lichen over a large part of the body, but most severely, perhaps, on the upper arms, on the flexure side of the elbows, and about the wrists. There was a rough symmetry in the grouping of the papules, which were sufficiently red and multitudinous to deserve the name of lichen agrius. There was not so much pruritus as a general heat or smarting; and as if to exhibit the eczematous or boiling-over process, which any form of skin-congestion may undergo, a large vesicle was developed on the front of each wrist among a crowd of angry papules. After a few days, the lichenous eruption desquamated on the arms as cleanly and decisively as if it were the sequel of a specific fever. On January 21st, small bullæ of pemphigus were seen on the back of both wrists and hands; presently also about the feet and ankles; and soon afterwards, very large bullæ formed on the legs, thighs and forearms. There were grave adynamic symptoms at the same time. By careful treatment and good nursing, the patient is now (Feb. 4th) pulling safely through. Now, how are we to interpret this chain of curious phenomena? Do they form a series of what Sir W. Gull calls 'nerve vagaries'—a succession of nerve-storms, one disturbance effacing as soon as possible what went before, as if in a hurry to display its own signs? Or, according to a less transcendental view, we may regard different elements of skin-texture as effected by certain morbid influences, one after the other; and the wonder is, that a person at such an advanced age should be able to survive the shock of so many cutaneous lesions."—*British Medical Journal.*

---

DR. HENRY B. WILBUR, superintendent of the State Idiot Asylum at Syracuse since its foundation, died suddenly at the asylum April 30th, aged sixty-three years. He was a native of Wilden, Mass., and a graduate of Amherst College, and was the pioneer educator ef idiots in this country, having established the first school for this purpose in his own house in 1848. He took charge of our experimental asylum at Albany in 1851, and three years later was appointed to the position at the State institution at Syracuse, which he filled with distinguished ability up to the time of his death.—*Boston Medical and Surgical Joarnal.*

---

CREDIT TO WHOM CREDIT IS DUE.—Philip Doddridge (1702–1751) spoke of nerve-stretching, and recommended it as a religious stimulant in his *Zeal and Vigor in the Christian Race*

> "Awake, my soul: STRETCH EVERY NERVE,
> And press with vigor on."

—*Glasgow Medical Journal.*

